# The effect of a midwifery continuity of care program on clinical competence of midwifery students and delivery outcomes: a mixed-methods protocol

**DOI:** 10.1186/s12909-024-05321-5

**Published:** 2024-03-26

**Authors:** Fatemeh Razavinia, Parvin Abedi, Mina Iravani, Eesa Mohammadi, Bahman Cheraghian, Shayesteh Jahanfar, Mahin Najafian

**Affiliations:** 1 Midwifery Department, Reproductive Health Promotion Research Center, Ahvaz Jundishapur University of Medical Sciences, Ahvaz, Iran; 2 Midwifery Department, Menopause Andropause Research Center, Ahvaz Jundishapur University of Medical Sciences, Ahvaz, Iran; 3 Midwifery Department, Menopause Andropause Research Center, Ahvaz Jundisahpur University of Medical Sciences, Golestan BLvd, Ahvaz, Iran; 4 Reproductive Health Promotion Research Center, Midwifery Department, Nursing and Midwifery School, Ahvaz Jundishapur University of Medical Sciences, Ahvaz, Iran; 5 Department of Nursing, Faculty of Medical Sciences, Tarbiat Modares University, Tehran, Iran; 6 Alimentary Tract Research Center, Clinical Sciences Research Institute, Department of Biostatistics and Epidemiology, School of Public Health, Ahvaz Jundishapur University of Medical Sciences, Ahvaz, Iran; 7 MPH Program, Department of Public Health and Community Medicine, Tufts University School of Medicine, Boston, USA; 8 Department of Obstetrics and Gynecology, School of Medicine, Ahvaz Jundishapur University of Medical Sciences, Ahvaz, Iran

**Keywords:** Continuity of care, Clinical competence, Mixed-methods, Midwifery students, Pregnancy outcomes

## Abstract

**Background:**

The midwifery continuity of care model is one of the care models that have not been evaluated well in some countries including Iran. We aimed to assess the effect of a program based on this model on the clinical competence of midwifery students and delivery outcomes in Ahvaz, Iran.

**Methods:**

This sequential embedded mixed-methods study will include a quantitative and a qualitative phase. In the first stage, based on the Iranian midwifery curriculum and review of seminal midwifery texts, a questionnaire will be developed to assess midwifery students’ clinical competence. Then, in the second stage, the quantitative phase (randomized clinical trial) will be conducted to see the effect of continuity of care provided by students on maternal and neonatal outcomes. In the third stage, a qualitative study (conventional content analysis) will be carried out to investigate the students’ and mothers’ perception of continuity of care. Finally, the results of the quantitative and qualitative phases will be integrated.

**Discussion:**

According to the nature of the study, the findings of this research can be effectively used in providing conventional midwifery services in public centers and in midwifery education.

**Trial registration:**

This study was approved by the Ethics Committee of Ahvaz Jundishapur University of Medical Sciences (IR.AJUMS.REC.1401.460). Also, the study protocol was registered in the Iranian Registry for Randomized Controlled Trials (IRCT20221227056938N1).

## Background

Providing quality services to pregnant women has been recommended to all countries to achieve the Millennium Development Goals (MDGs) (Goals 3, 4 and 5) [1]. There are different care methods to maintain maternal and neonatal health during pregnancy and postpartum [1]. One of these care models is continuity of care that can be provided by a midwife or an obstetrician.

Midwifery continuity of care is a relationship-based care provided by a midwife who can be supported by one to three more midwives. They provide planned care for a woman during pregnancy, labor, birth, and the early postpartum period up to 6 weeks after delivery [2].

Continuity of midwifery care has become a global effort to enable women to have access to high-quality maternity care and delivery services [3]. As a result, many service providers today are transitioning to a continuous care model [4], and they have considered continuous care to be necessary for realizing women's rights [5]. Also, continuous midwifery care is known as the gold standard in maternity care to achieve excellent results for women [5, 6]. In order to strengthen midwifery services to achieve global health goals in 2015, the World Health Organization (WHO) proposed a midwife-led continuous care model [7].

Countries use different midwifery care models. In Iran, for example, primary health services that are specific to pregnant mothers are provided in public health centers by midwives working in the network system and in compliance with the level of services and the referral system [8].

In general, midwifery continuous care not only has an important impact on a wide range of health and clinical outcomes for mothers and neonates but also brings about economic consequences for the health system [2, 9]. This care model is useful for healthcare professionals as well [10], and it has improved the job satisfaction of midwives [11]. The midwife is the main guide in planning, organizing and providing care to a woman from the beginning of pregnancy to the postpartum period [12]. In 2011, in order to increase job motivation and satisfaction, promote retention of the midwifery workforce [13], and alleviate the shortage of workforce at the international level [14], the Nursing and Midwifery Advisory Center recommended using midwifery students (at the bedside and to perform midwifery work) to overcome this problem.

Providing high quality care requires enhancing the clinical competence of the professionals [4]. There is a close relationship between the concept of patient care quality and clinical competence. Therefore, clinical competence is of unique importance in midwifery practice [15]. As a result, in order to achieve quality patient care, midwifery professionals need to train students to become workforce with clinical competence in order to provide quality care in the health system. WHO defined clinical competence as a level of performance that demonstrates the effective application of knowledge, skills, and judgment [16].

A previous study showed that clinical competence of midwives plays an important role in managing the process of providing care, achieving care goals, and improving the quality of midwifery services [17]. In other words, the graduates of this field must have an acceptable level of clinical and professional skills in performing midwifery duties so that the health of mothers, children, and ultimately the community can be improved.

In Iran, prenatal care and the care during labor, delivery and postpartum are not continuous, and a new health provider may take the responsibility of care at any stage. This fragmented care may negatively affect the pregnancy outcomes and increase the rate of cesarean section [18]. Furthermore, the results of some studies in Iran indicate that the clinical competence obtained by midwifery students is far from optimal and that they do not acquire the necessary skills and abilities at the end of their studies [19]. Farrokhi et al. showed that the performance quality of 70% of midwives is average, and only 18.5% of them have good quality performance [20]. Several factors play a role in acquiring, maintaining and improving clinical competence [21]. There are a number of solutions that can increase the clinical competence of midwifery students, and one is the use of different care models such as the continuity of care model. The continuity of care model allows students to develop their midwifery knowledge, skills, and values individually [22]. Despite the strong foundation of midwifery in Iran, midwifery care models have not yet been tested. Some studies have reported that the quality of services provided during pregnancy, delivery and after delivery in Iran is poor to moderate. Also, these studies emphasize the necessity of a paradigm shift for better quality care and greater satisfaction of mothers, and they consider lack of continuity of care as the reason for the increase in unnecessary cesarean sections [23–25]. Moreover, the lack of qualified and experienced workforce has led to low quality health services, including midwifery care, and an increase in the economic burden of health. In Iran, no study has yet been conducted to investigate the effect of the midwifery continuity of care model on the students’ clinical competence and pregnancy outcomes. Given the importance of this topic, using a mixed-methods study design, we aimed to assess the effect of a midwifery continuity of care program on the clinical competence of midwifery students and pregnancy outcomes in Ahvaz, Iran.

### Specific objectives

1. To determine the effect of midwifery continuity of care program on the clinical competence of midwifery students.
2. To determine the effect of a midwifery continuity of care program provided by midwifery students on pregnancy outcomes.
3. To explain the perception of midwifery students and mothers about the use of the midwifery continuity of care program provided by midwifery students.

## Methods/design

### Study design

**Fig. 1:**
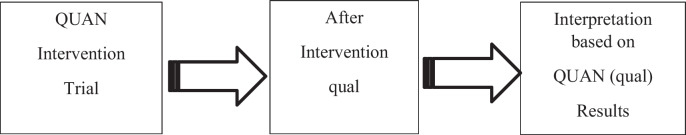
Sequential and embedded mixed-methods design

This sequential embedded mixed-methods study will include a quantitative phase and a qualitative one. A mixed (embedded) experimental design involves the collection and analysis of quantitative and qualitative data by the researcher and the integration of the information into an experimental study or intervention trial. This design adds qualitative data to an experiment or intervention to integrate the personal experience of research participants. Therefore, the qualitative data are converted into a secondary source of data embedded before and after the test. Qualitative data is added to the experiment in differrent ways, including: before the experiment, during the experiment, or after the experiment [26, 27]. Embedded mixed-methods studies that are qualitative followed by quantitative are used to understand the rationale for the results and receive feedback from participants (to confirm and support the findings of the quantitative studies) [27]. In the first stage of this study, a questionnaire for assessing midwifery students’ clinical competence will be created based on the midwifery curriculum of Iran and a review of seminal texts of midwifery. Then, the effect of continuity of care provided by midwifery students on maternal and neonatal outcomes will be assessed in a randomized clinical trial. In the third stage, a qualitative study will be carried out to investigate the perception of students and mothers. Finally, the results of the quantitative and qualitative phases will be integrated (Fig. 1).

### First stage: questionnaire development

This questionnaire will be developed based on midwifery curriculum and a comprehensive and systematic search (with no time limit) in English and Persian databases (Web of Science, Embase, Scopus, ProQuest, Google scholar, Magiran, SID).

### Tool design

There are four steps in tool development:

1. Choosing a conceptual model to show aspects of clinical competence in the measurement process
2. Explaining the purpose of the tool
3. Designing the route map
4. Developing the tool (use of methods, classification of objects, rules and procedures for scoring tools) [28].

### Answer to the objects

A 1 to 4-point Likert scale will be used for scoring [29].

### Content validity

To ensure the selection of the most important and correct content (necessity of the case), the content validity will be assessed. Also, to ensure that the instrument items are designed in the best way to measure the content, the content validity index will be calculated [30].

### Reliability

Reliability will be evaluated using internal consistency (Cronbach's alpha coefficient ≥ 0.7) and stability (test-re-test ≥ 0.74) by piloting the questionnaire on 20 midwifery students [31].

### Second stage: quantitative phase

A randomized controlled clinical trial will be conducted in this phase of research to examine the effect of the continuous care program of midwifery students on their clinical competence and pregnancy outcomes.

### Sample size

According to the study objective and previous study results [32] with α = 0.01, β = 0.1, p_1_ = 0.51 and p_2_ = 0.021, the sample size will be *n* = 23. Considering a 20% dropout rate, the final sample size will be 58 women (29 women in each group).

$$\begin{array}{l}n=\frac{{\left({z}_{1-\alpha /2}+{z}_{1-\beta }\right)}^{2}*\left[{p}_{1}\left(1-{p}_{1}\right)+{p}_{2}\left(1-{p}_{2}\right)\right]}{\left({p}_{1-}{p}_{2}\right)^{2}}\\ \mathrm{Z}_{1-\mathrm{\alpha }/2}=2.57\\ \begin{array}{l}\mathrm{Z}_{1-{\mathrm\upbeta} }=1.28\\ {\left(2.57+1.28\right)}^{2}*\left(0.55\left(0.45\right)+0.021\left(0.979\right)\right)/{\left(0.529\right)}^{2}=23\end{array}\end{array}$$

### Data collection

This phase of the randomized clinical trial will be conducted with the participation of 58 undergraduate midwifery students at their 7th and 8th semesters. The students will be divided randomly to intervention (continuous care) and control (routine care) groups providing care to 58 pregnant women in six health centers and two hospitals (Sina and Razi) in Ahvaz city, southwest of Iran.

The study will begin after receiving the approval of the Ethics Committee of Ahvaz University of Medical Sciences and registering the study in the Iranian Registry for Randomized Clinical Trials. Inclusion criteria will be willingness to participate in the study.

### Randomization

To implement the intervention, the students will be divided into two intervention (providing continuous care for pregnant women) and control (providing standard care for pregnant women) groups. Allocating students will be done using permuted block randomization technique with a block size of four and an allocation ratio of 1:1. Five blocks of 4 pieces and 3 blocks of 3 pieces will be extracted randomly using WIN PEPI software. In each block of 4, 2 students will be in control and 2 will be in intervention group. Also, in each block of 3 students, 1 student will be in control and 2 will be in intervention group, and the arrangement of each person is random. To prevent contamination, first the control group will provide routine care, and then the intervention group will conduct continuity of care for pregnant women. Mothers are randomly selected based on the hospital where they will give birth. As a result, Razi Hospital will be the control group and Sina Hospital will be the intervention group.

### Intervention

Women who meet the inclusion criteria will be recruited in the study using a non-probability convenience sampling method. Women in the intervention group will be included in the study after their first pregnancy visit (6–10 weeks of gestation) and will receive continuous care by midwifery students. Women in the control group will receive the usual and routine care, and will be included in the study at the time of delivery. They will have a gestational age of more than 37 weeks based on the inclusion criteria of the study. Their delivery will be performed by midwifery students who will follow them up until six weeks after delivery.

At first, the necessary training will be given by the lead researcher (FR) to the students in orientation sessions held for both groups separately. In the intervention group, each midwifery student as the main midwife will be responsible for taking care of two or three pregnant women and will be the back-up midwife for two other pregnant women (under the supervision of other students). The lead researcher will create a group in WhatsApp with the participation of students in the intervention group, and they can communicate with each other and the researcher. Also, the midwifery students will be directly and indirectly under the supervision of a qualified person (lead researcher). Another WhatsApp group will be created for the women of the intervention and control groups (to facilitate communication between the researcher and the women). Two midwifery students will be introduced to each pregnant woman in the intervention group (as a main midwife and a backup midwife). If the main midwife is not available, the woman will be in contact with the backup midwife. The backup student will meet the woman at least once and will be introduced to her.

### Instruments

All students and pregnant women participating in this study will complete a demographic questionnaire. A checklist will be provided for collecting data during prenatal care, labor, and delivery.

Also, the midwifery students will complete the clinical competency questionnaire at the beginning and end of the study.

Care will be provided and recorded by the main student according to the pregnancy care protocol. Also, danger signs will be taught to the students according to the national protocol, and emergencies will be handled by the midwifery student under the supervision of the lead researcher. Admission to hospital will be arranged by the student, and all information will be recorded. Pregnancy, labour and delivery, postpartum, and newborn checklist will be completed. Students will complete a demographic and obstetric questionnaire that includes questions about age, education, occupation, gravidity, parity, abortions, live and dead children, last contraceptive method, intended and unintended pregnancies, last menstrual period (LMP), gestational age, date of birth, body mass index (BMI), previous pregnancy and childbirth records, high-risk behavior of the mother and father, current history of special care, test and ultrasound results, and participation in childbirth preparation class. Also, the following data will be recorded in the labor and delivery and post-partum checklist: checking the conditions of labor according to the partograph, length of labor, need for induction and the method used type of delivery, examination of perineal trauma, postpartum bleeding, and examination of the condition of the mother up to 6 weeks after delivery. In addition, the amount of bleeding will be checked visually and by measuring the level of hemoglobin and hematocrit. Apgar score of the newborn will be recorded (in infant checklist) in minutes 1 and 5. Also, the newborn’s hospitalization status, breastfeeding and anthropometric indices will be recorded.

### Follow-up

The students in the intervention group will start prenatal care < 20 weeks of gestation. At least five round of prenatal care will be provided by each student according to national guidelines for each pregnant woman. Pregnant women can communicate with their in-charge students in non-emergency cases from 8:00 a.m. to 23:00 p.m. and in emergency cases 24 h a day, all days a week. All reports will be recorded by the students. During labor and delivery, the student and the lead researcher will be present at the mother's bedside. In case of natural vaginal delivery (NVD), delivery will be done by a student midwife under the supervision of the researcher. In case of cesarean delivery (CS), a student will be present at the patient's bedside. Postpartum care will be provided by midwifery students in both groups (intervention and control). Each student will be at the mother's bedside for two hours after delivery. The conditions of labor, delivery, and the neonate will be recorded by the student in the relevant form. Also, the mother will be followed up by telephone for up to 6 weeks after delivery (postpartum). The clinical competency questionnaire will be completed by students before and after the intervention.

### Inclusion criteria

Inclusion criteria for midwifery students will be: studying at the seventh and eighth semester and willingness to participate in the study.

Inclusion criteria for service recipients (pregnant women) will be: age 18 – 40 years, Iranian nationality, singleton pregnancy, low risk pregnancy, and gestational age < 20 wks.

### Exclusion criteria

Exclusion criteria will be: history of psychiatric disorders, previous caesarean section, use of alcohol and tobacco, or having a disease that requires prenatal care by a specialist.

### Primary outcome

Clinical competence of midwifery students.

### Secondary outcome

Mode of delivery, length of labor stages, the need to induction, postpartum bleeding first and fifth minute Apgar score, admission of neonate to the neonatal intensive care unit, breastfeeding initiation, and exclusive breastfeeding up to 6 weeks postpartum.

### Data analysis

Statistical analyses will be done using SPSS version 26.0 (SPSS, Inc., Chicago, IL, USA). The independent t-test and Chi-square tests will be used for continuous data and categorical data, respectively. ANCOVA test will be used to eliminate the influence of confounding variables. The effect size will be calculated. A 95% confidence interval (CI) and *p* values will be reported. *P*-values less than 0.5 will be considered statistically significant.

### Third step of research: qualitative study

This phase will be a qualitative study using conventional content analysis.

### Sample size

Purposeful sampling will be used in this study [33]. Sampling will continue until data saturation [34], i.e., no new information or data about a class or relationships between classes is revealed.

### Data collection

This phase of the study is a conventional qualitative content analysis [35] aimed at examining the perceptions of midwifery students and mothers receiving continuous care. The researcher will conduct in-depth, semi-structured interviews with open-ended questions with students and mothers in the group of the continuous care program. All interviews will be done by the lead researcher who is qualified in qualitative research method. The interview will start with a general and open question such as: “Please tell me about your experiences or feelings about participating in the continuous midwifery care program. How did you feel about participating in this program?” Then, in-depth exploratory questions will be asked based on their answers (e.g., what do you mean? Why? Can you elaborate on that? Can you give me an example so I can understand what you mean?). All interviews will be recorded with the participants' consent. Paralinguistic features, such as mood and features of the participants, including tone of voice, facial expressions, and their posture, will be recorded by the researcher during the interview [35].

The data will be analyzed based on Granheim and Lundman's 2004 content analysis approach [36].

Interviews will be transcribed at the end of each interview. Data analysis begins with a careful study of all data so that the researcher can immerse herself in the data and gain an overview. Interviews will be transcribed verbatim. Key concepts will be highlighted and codes will be extracted. Then the first interpretations will be made and analyzed. Labels emerge for codes that represent more than one key concept and are usually taken directly from the text and become the initial coding map. Then the codes are placed in the category based on their similarity. Then, definitions will be created for each category, subcategory and code. When reporting findings, examples of each code and data category will be provided [35].

### Inclusion criteria

Inclusion criteria for midwifery students will be: studying at the seventh or eighth semester, willingness to participate in the study.

Inclusion criteria for service recipients (pregnant women) will be: receiving continuous care provided by the student, willingness to participate in the study, and being able to communicate.

### Data analysis

The qualitative study and interview data will be analyzed based on the content analysis approach of Granheim and Lundman 2004 [36] as follows:

1. Reading and re-reading the interviews after completion of each interview
2. Selection of the unit of analysis
3. Determination of semantic units
4. Coding
5. Classification
6. Extraction of information content

In the first step, the data is converted into text format. As soon as possible after the interview, the interview will be typed verbatim. Then the whole text will be read several times to get a general understanding of the content of interview. Each meaning unit will be converted into condensed meaning units and then coded. The Codes will be classified into subcategories and categories based on their common characteristics. Finally, the content of the categories will be revealed, taking into account their hidden meaning [36].

### Trustworthiness

Five criteria of will be used to increase data trustworthiness according to Lincoln & Guba [37]. These include: 1. Credibility, 2. Dependability, 3. Confirmability, 4. Transferability, 5. Authenticity.

Credibility of the data will be ensured by continuous engagement of the researchers with the subject, member checks, and external checks. Dependability will be ensured by relying on the insight of external observers. In order to increase the confirmability, data will be accurately recorded and reported. Also, transferability will be ensured by presenting the research process accurately, clearly and purposefully, which includes purposive sampling and presenting the research results to a number of people with the same profile of the participants who did not participate in the research. Finally, authenticity will be guaranteed by continuous reflection on information, long-term presence of the researcher, interview recording, writing, and reporting of findings.

### Combining qualitative and quantitative phases

**Fig. 2:**
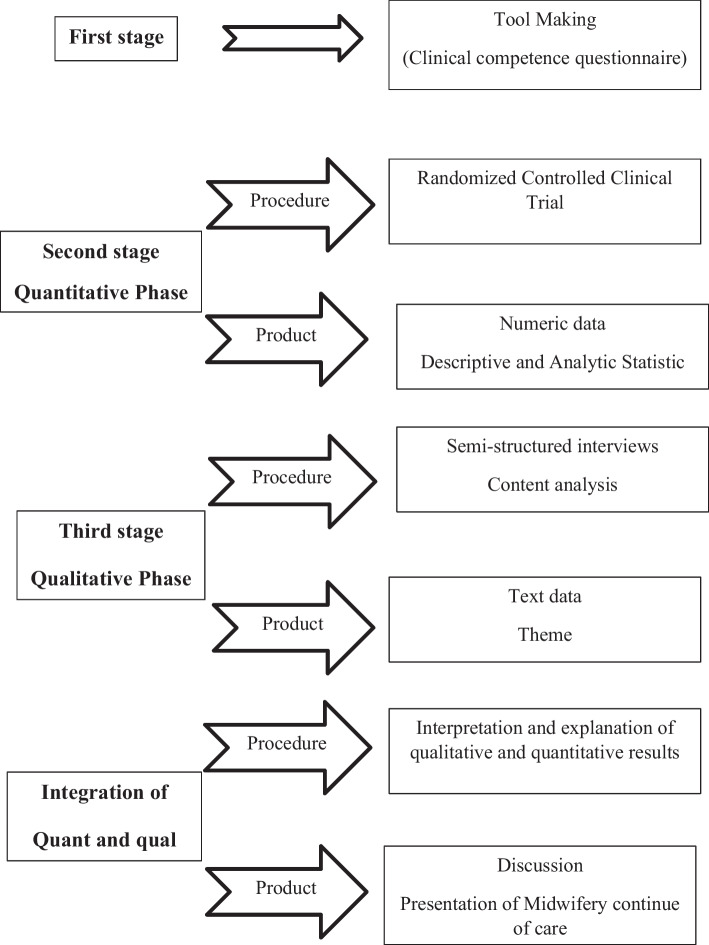
Study diagram

Data combination will be done using data integration strategies. The integration or combination of data starts from quantitative data analysis. Then qualitative data is collected by interview. In fact, the qualitative study is a secondary source of embedded data in the collection of experimental test data (continuous care) after the quantitative study. In this research, in order to understand the results of the RCT, the views of the participants will be unified in order to get a correct understanding of the intervention (implementation of the continuity of care model by the students) from the mothers' and students' point of view (Fig. 2).

### Study status

The development of the evaluation tools was made. Also, sampling the quantitative phase of the study and the basic of the program are in process (Table 1).

**Table 1:**
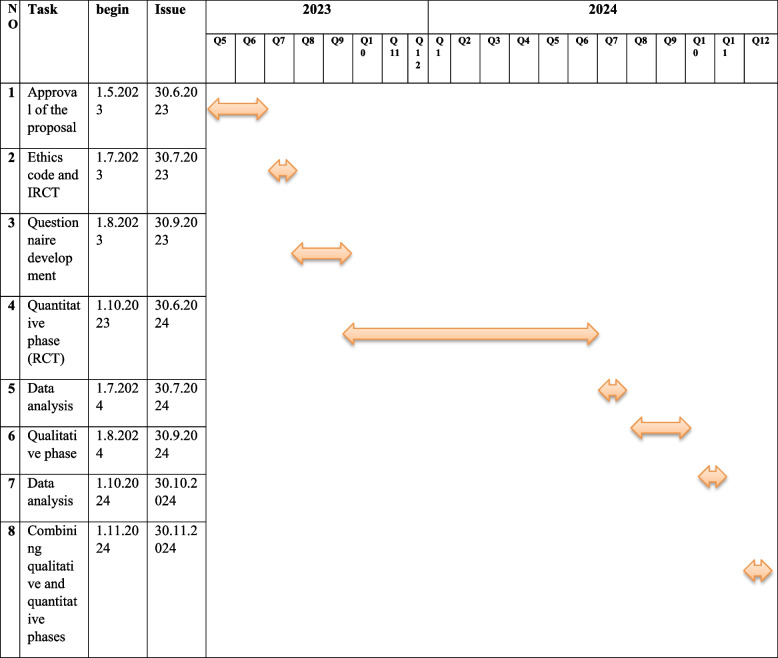
Flowchart of the study, with detailed issues and tasks

## Discussion

This is the first mixed-methods study to be conducted in Iran investigating the effect of a midwifery continuity of care program on clinical competence of midwifery students and pregnancy outcomes. According to the recommendations of the WHO, midwifery continuity of care should be adopted in order to increase the quality of pregnancy care as well as the satisfaction of pregnant women and service providers [7]. Contrary to the recommendation of WHO, the continuous care program is neither implemented in Iran's health system nor included in the midwifery curriculum. The results of this study can help health planners and policy makers to implement high quality midwifery care program based on global recommendations.

The study has several strengths. The use of a mixed-methods study design (combination of quantitative and qualitative approaches) in contrast to the separate use of quantitative and qualitative studies provides a better understanding of the research questions [38]. In embedded design, one type of data collection (quantitative or qualitative) plays a supporting and essential role for another type. As a result, the embedded mixed-methods technique in the qualitative phase after designing the intervention will be used to receive feedback from the participants to confirm and support the findings of quantitative phase [39]. Also, interviews with mothers and midwifery students in the intervention group can reflect their positive and negative experiences of this program. Considering that Iran's healthcare system lacks continuous midwifery care, the findings of this research can be effectively used in providing conventional midwifery services in public centers and in midwifery education.

Considering that this care model will be implemented for the first time in Iran's midwifery education and healthcare system, there may be two possible limitations in this study: lack of infrastructure and interference with other educational programs.

## Data Availability

All the data that will be obtained will be published in the next article after the implementation of the study.

## Abbreviations

BMI: Body mass index
CS: Cesarean section
LMP: Last menstrual period
MDGs: Millennium Development Goals
NVD: Natural vaginal delivery
WHO: World Health Organization
